# Supplementary material for: CPPs to the Test: Effects on Binding, Uptake and Biodistribution of a Tumor Targeting Nanobody
Source: Pharmaceuticals (Basel). 2021 Jun 23;14(7):602. doi: 10.3390/ph14070602 (PMC8308549; doi:10.3390/ph14070602)
Supplement: Supplementary file 1 [file pharmaceuticals-14-00602-s001.zip › pharmaceuticals-1228542-supplementary.pdf]

Appendix A: Supplementary tables.

Table S1. Names and abbreviations and sequences of the CPPs used. All peptides were C-terminally amidated (-NH_2).

Name (Abbreviation)	Peptide Sequence
L-nonaarginine (R9)	RRRRRRRRR-NH2
D-nonaarginine (r9)	rrrrrrrrr-NH2
L-penetratin (Pen)	RQIKIWFQNRRMKWKK-NH2
D-penetratin (pen)	rqikiwfqnrrmkkwkk-NH2
Tat	GRKKRRQRRRPQ-NH2
hLF	KCFQWQRNMRKVRGPPVSCIKR-NH2

Table S2. Theoretical and found masses of the various conjugates as measured with ESI-ToF mass spectrometry.

Compound	Theoretical Mass (Da)	Found Mass (Da)	Difference Theoretical-Found
7D12-DTPA	18,354.9	18,342.9	12
7D12-DTPA-r9	17,256.8	17,227.9	28.9
7D12-DTPA-pen	18,079.9	18,051.1	23.8
7D12-Atto532	18,606.5	18,595.0	11.5
7D12-Atto532-R9	17,508.4	17,497.3	11.1
7D12-Atto532-Pen	18,331.5	18,319.8	11.7
7D12-Atto532-Tat	17,649.6	17,638.5	11.1
7D12-Atto532-hLF	1803.0	18,790.2	12.8

Table S3. Biodistribution results (%ID/g) 4 h after injection.

sample	7D12	7D12 Block	7D12-Pen	7D12-Pen Block	7D12-r9	7D12r9 Block
A431	9.63 ± 2.34	2.69 ± 0.51	4.56 ± 1.01	4.06 ± 2.94	1.98 ± 0.39	1.69 ± 0.34
SKOV3	3.85 ± 3.63	0.81 ± 0.63	2.45 ± 0.61	0.99 ± 0.28	1.33 ± 0.36	0.72 ± 0.09
blood	0.28 ± 0.08	0.09 ± 0.03	0.29 ± 0.15	0.25 ± 0.10	0.31 ± 0.13	0.41 ± 0.48
colon	0.48 ± 0.04	0.32 ± 0.09	0.51 ± 0.07	0.52 ± 0.13	0.28 ± 0.05	0.55 ± 0.07
duodenum	0.47 ± 0.03	0.25 ± 0.06	0.45 ± 0.09	0.73 ± 0.21	0.51 ± 0.04	0.59 ± 0.21
heart	0.22 ± 0.06	0.11 ± 0.02	0.26 ± 0.06	0.27 ± 0.04	0.25 ± 0.03	0.35 ± 0.05
lung	0.54 ± 0.12	0.36 ± 0.07	4.36 ± 2.75	4.97 ± 2.07	1.03 ± 0.25	1.57 ± 0.47
muscle	0.11 ± 0.01	0.11 ± 0.04	0.14 ± 0.02	0.13 ± 0.04	0.10 ± 0.04	0.13 ± 0.02
pancreas	0.21 ± 0.03	0.13 ± 0.02	0.31 ± 0.09	0.22 ± 0.03	0.14 ± 0.09	0.26 ± 0.15
spleen	0.69 ± 0.08	0.31 ± 0.04	3.79 ± 0.75	5.67 ± 2.60	4.71 ± 1.00	4.86 ± 0.66
stomach	0.34 ± 0.04	0.25 ± 0.03	0.47 ± 0.06	0.47 ± 0.09	0.44 ± 0.25	0.43 ± 0.11
liver	1.85 ± 0.30	0.70 ± 0.11	24.5 ± 2.03	17.44 ± 8.98	47.99 ± 5.98	46.78 ± 24.56
kidney	216.71 ± 25.05	86.63 ± 44.55	124.92 ± 7.95	66.48 ± 33.80	40.35 ± 3.37	30.31 ± 2.29

Table S4. Biodistribution results (%ID/g) 24 h after injection.

sample	7D12	7D12 Block	7D12-Pen	7D12-pen Block	7D12-r9	7D12-r9 Block
A431	4.79 ± 0.41	1.75 ± 0.09	2.75 ± 0.71	1.66 ± 0.42	1.16 ± 0.15	1.05 ± 0.14
SKOV3	2.44 ± 1.63	0.51 ± 0.14	2.40 ± 1.34	0.79 ± 0.30	0.85 ± 0.45	1.90 ± 2.44
blood	0.07 ± 0.05	0.02 ± 0.01	0.12 ± 0.08	0.10 ± 0.03	0.09 ± 0.05	0.18 ± 0.13
colon	0.34 ± 0.05	0.21 ± 0.02	0.35 ± 0.03	0.38 ± 0.04	0.14 ± 0.03	0.23 ± 0.04
duodenum	0.29 ± 0.07	0.18 ± 0.03	0.34 ± 0.08	0.56 ± 0.19	0.24 ± 0.02	0.31 ± 0.05
heart	0.11 ± 0.03	0.07 ± 0.00	0.16 ± 0.01	0.20 ± 0.05	0.10 ± 0.01	0.12 ± 0.02
lung	0.21 ± 0.04	0.17 ± 0.03	1.78 ± 1.07	1.53 ± 0.71	0.32 ± 0.03	0.50 ± 0.16
muscle	0.09 ± 0.03	0.07 ± 0.01	0.10 ± 0.03	0.13 ± 0.04	0.08 ± 0.05	0.05 ± 0.01
pancreas	0.16 ± 0.05	0.14 ± 0.06	0.20 ± 0.04	0.31 ± 0.10	0.10 ± 0.02	0.26 ± 0.14

spleen	0.88 ± 0.53	0.36 ± 0.09	3.48 ± 0.61	7.54 ± 3.34	3.50 ± 0.82	4.67 ± 0.35
stomach	0.22 ± 0.04	0.18 ± 0.02	0.28 ± 0.05	0.42 ± 0.19	0.23 ± 0.12	0.26 ± 0.08
liver	1.17 ± 0.56	0.50 ± 0.06	17.51 ± 1.57	13.73 ± 4.61	33.69 ± 2.56	32.38 ± 10.99
kidney	174.65 ± 15.88	64.22 ± 11.41	93.76 ± 14.69	57.74 ± 8.27	30.33 ± 2.20	18.91 ± 3.27

Appendix B: Supplementary figures.

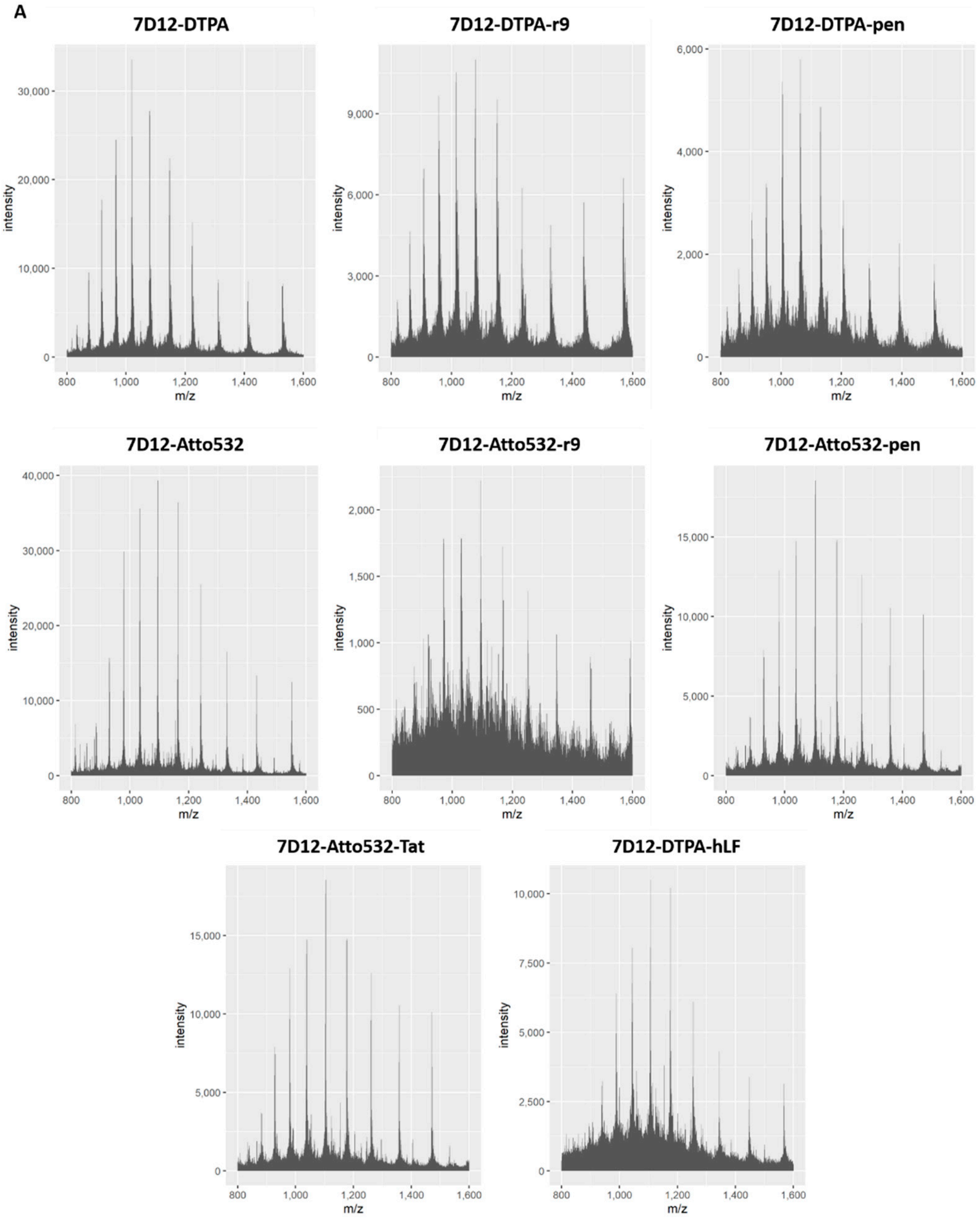

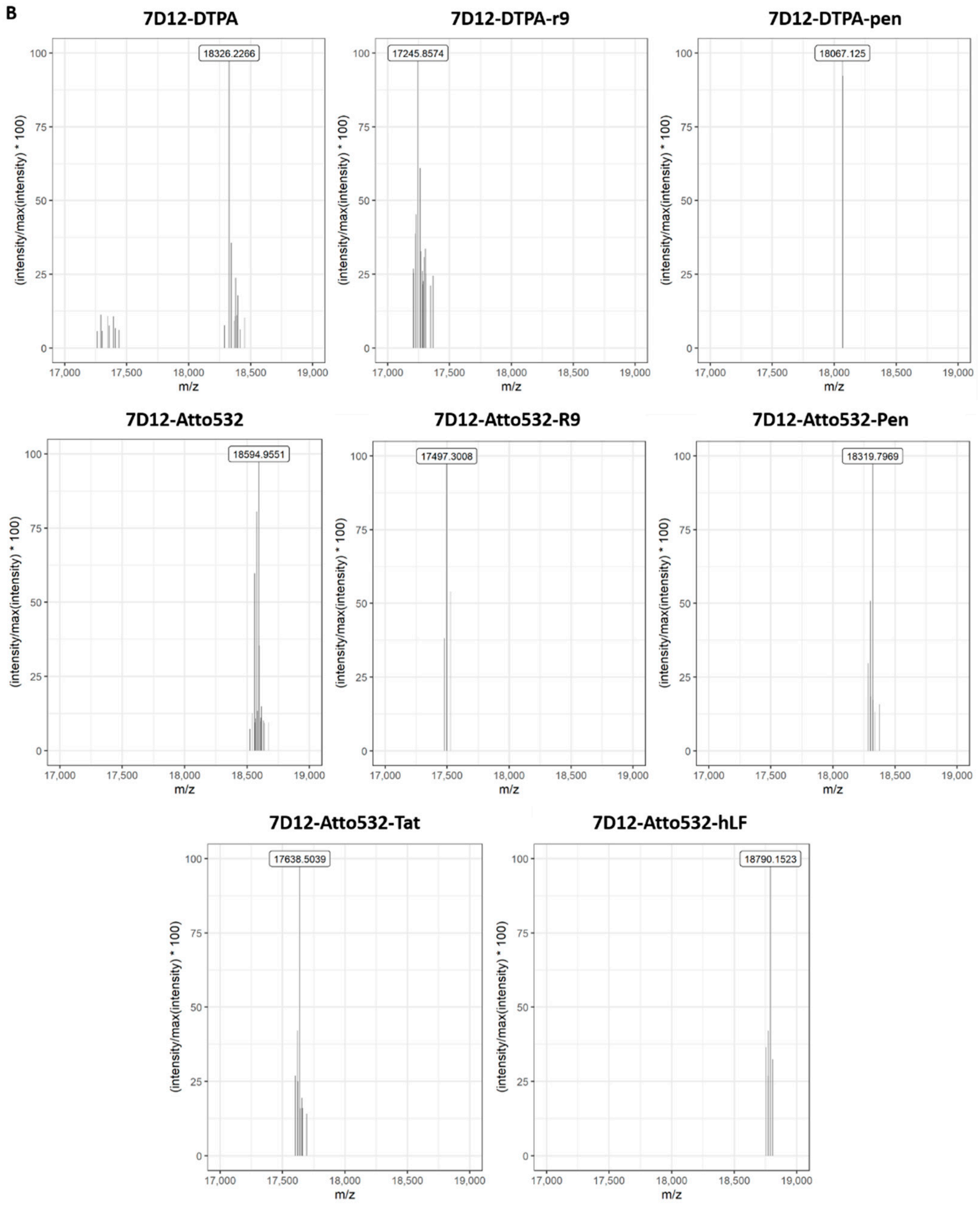

Figure S1. (**A**) m/z spectra of 7D12-Atto532 and CPP conjugates and 7D12-DTPA and CPP conjugates. (**B**) Deconvoluted masses of 7D12-Atto532 and CPP conjugates and 7D12-DTPA and CPP conjugates.

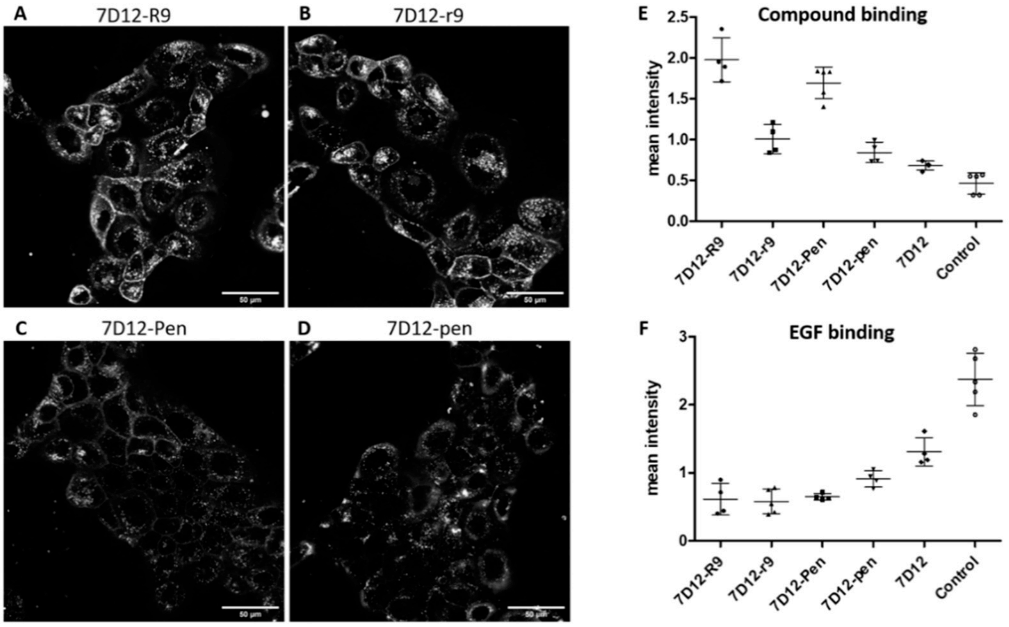

Figure S2. (**A**–**D**) Confocal microscopy images of live A431 cells incubated with 2 μM 7D12-Atto532-R9 (**A**), 7D12-Atto532-r9 (**B**), 7D12-Atto532-pen (**C**), 7D12-Atto532-Pen (**D**). (**E**) Quantification of conjugate binding in a sequential incubation experiment as described for Figure 3. (**F**) Quantification of EGF binding in a sequential incubation experiment as described for Figure 3. Mean intensity in arbitrary units. Each dot corresponds to one field of view and error bars indicate SD. Two fields of view were quantified for each of two independent experiments.

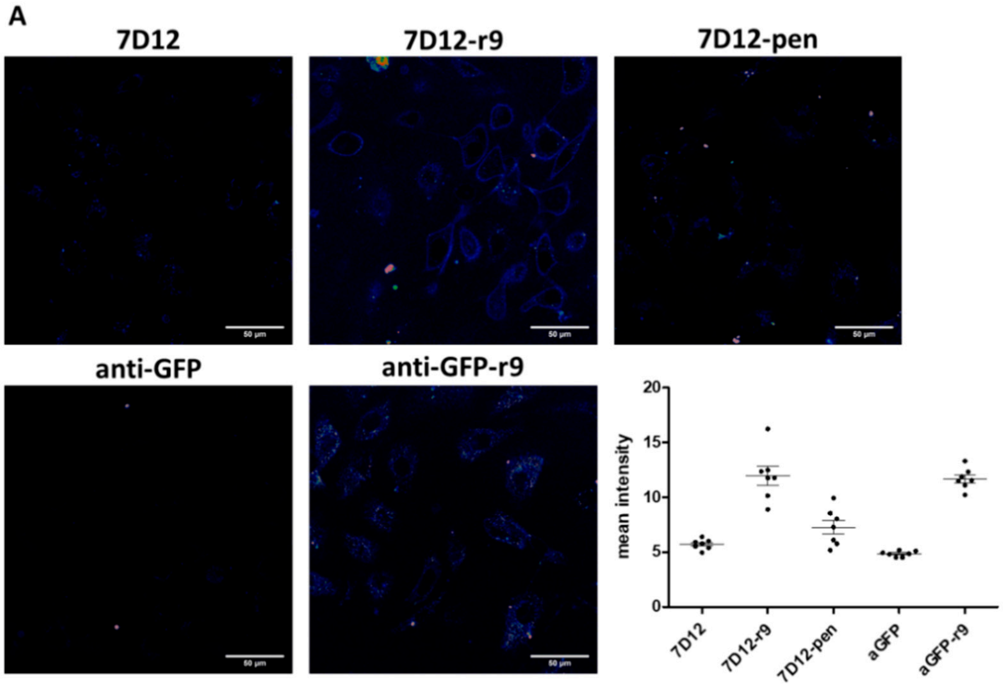

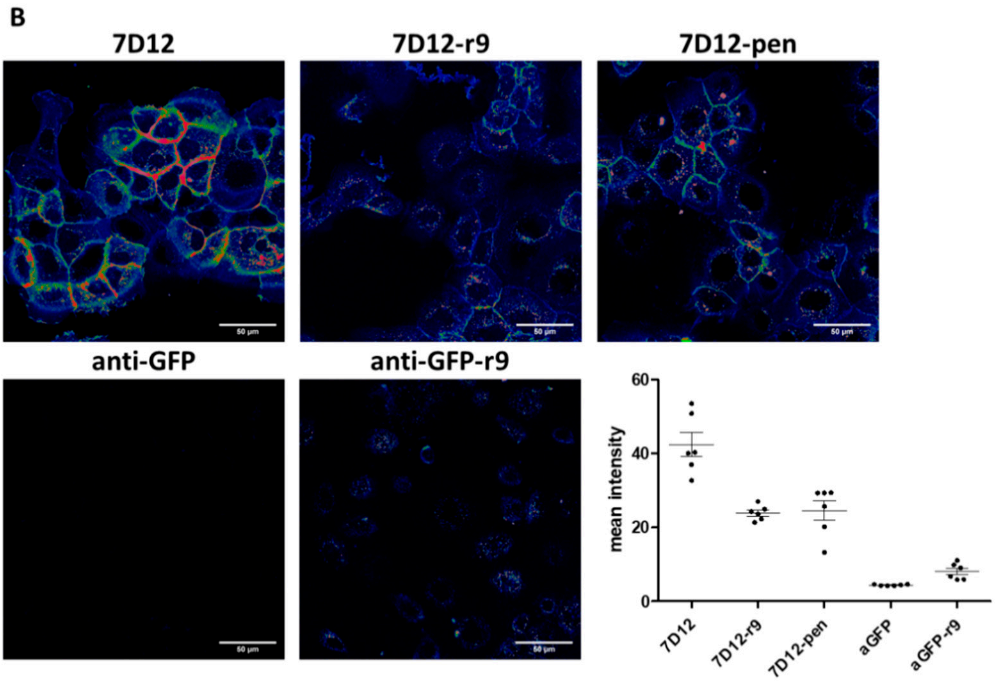

Figure S3. (**A**) Confocal microscopy images of live SKOV3 cells incubated with 0.5 μM 7D12-Atto532, 7D12-Atto532-r9, 7D12-Atto532-pen and control anti-GFP nanobody and its conjugate, anti-GFP-r9. (**B**) Confocal microscopy images of live A431 cells incubated with 0.5 μM 7D12-Atto532, 7D12-Atto532-r9, 7D12-Atto532-pen and control anti-GFP nanobody and its conjugate, anti-GFP-r9. Mean intensity in arbitrary units. Each dot corresponds to one field of view and error bars indicate SD. Two fields of view were quantified for each of two independent experiments.

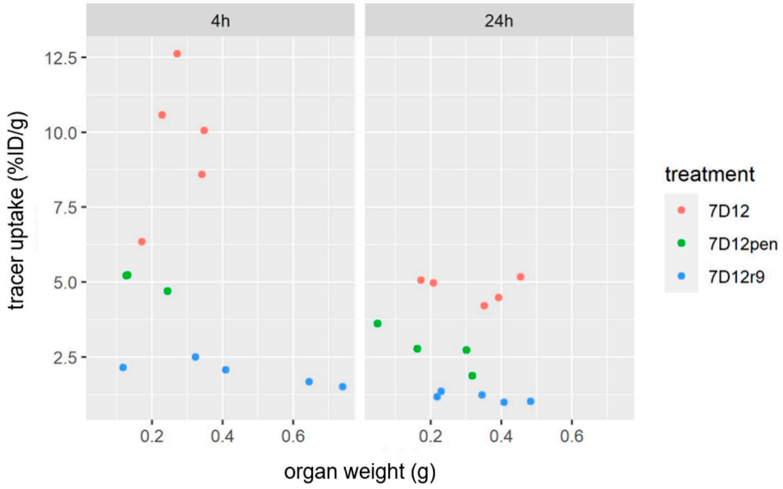

Figure S4. Correlation between tumor weight and absolute tracer uptake (not corrected for tissue weight).

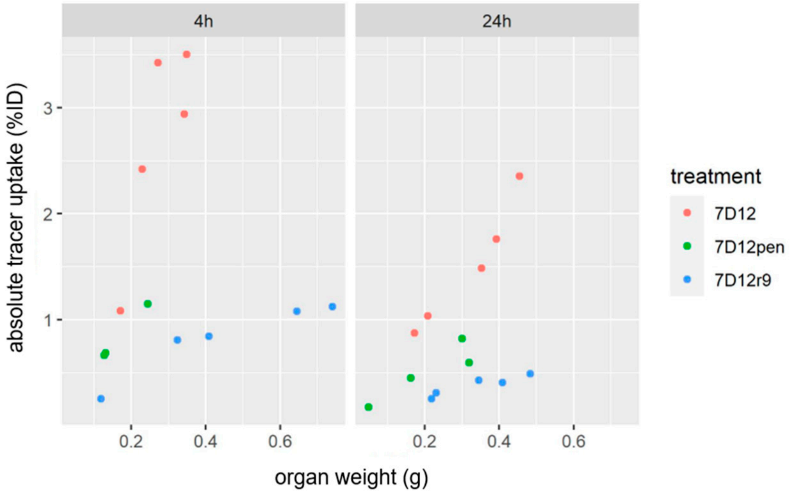

Figure S5. Correlation between tumor weight and relative tracer uptake.
